# Absent or not invited? A multilevel qualitative study of patients and healthcare professionals’ perspectives on barriers and enablers of outpatient attendance in Southern Denmark

**DOI:** 10.1186/s12913-026-14533-y

**Published:** 2026-04-27

**Authors:** Daria Morgounova Schwalbe, Luna Richardt, Morten Sodemann, Nina Høy Chodkiewicz, Nikoline Grønbech Grøtli, Karin Yde Waidtløw, Bente Mertz Nørgård, Maria Iachina, Ken Lund, Jette Ammentorp

**Affiliations:** 1 Center for Research in Patient Communication, Department of Clinical Research, Odense University Hospital and University of Southern Denmark, Odense, Denmark; 2 Center for Clinical Epidemiology, Odense University Hospital, Odense, Denmark; 3 Research Unit of Clinical Epidemiology, Department of Clinical Research, University of Southern Denmark, Odense, Denmark; 4 Department of Infectious Diseases and the Migrant Health Clinic, Odense University Hospital, Odense, Denmark; 5 Research Unit of Infectious Diseases, Department of Clinical Research, University of Southern Denmark, Odense, Denmark

**Keywords:** Non-attendance, Outpatient appointments, Participatory action research (PAR), Ethnography, Communication, Structural barriers, Healthcare system

## Abstract

**Background:**

Missed outpatient appointments are commonly framed as a problem of patient compliance and responsibility (non-attendance). This paper challenges that assumption by demonstrating how communication practices and organisational structures shape experiences of care and access in Southern Denmark. Drawing on participatory action research, the study explores barriers and enablers of attendance from the perspectives of patients and healthcare professionals and identifies potential measures to reduce missed appointments.

**Method:**

We adopt a multilevel participatory approach. As part of participatory action research, we conducted two workshops and two focus groups, working closely with Danish and immigrant patients, professional interpreters, health care administrative professionals, and technical specialists to explore barriers and enablers of outpatient attendance. Participants were purposively recruited through patient and professional networks and via digital platforms, including LinkedIn and FB. We complemented the study with ethnographic observations and semi-structured interviews with head nurses and medical secretaries in orthopaedic surgical departments at two regional hospitals. Seven patients were additionally interviewed. Field notes, workshop transcripts, and interview data were analysed using reflexive thematic analysis to identify patterns of meaning, and barriers and enablers of attendance, across participant groups and organisational contexts.

**Results:**

The study identified a range of interrelated barriers to attendance grouped into eight overarching themes: (1) structural barriers related to digital and logistic inequalities, (2) systemic and organisational barriers, (3) communication barriers, (4) psychological or illness-related barriers, (5) cultural and language barriers, (6) emotional barriers; (7) relational barriers, and (8) barriers related to physical space and wayfinding. Continuity of care and clear and direct communication were identified as factors contributing to attendance. Suggestions for improving attendance included strengthening cross-departmental collaboration, adopting more person-centred communication, and implementing multilingual communication tools.

**Conclusions:**

Non-attendance may stem from communication failures, systemic limitations, and structural barriers within the healthcare system, rather than solely from poor patient compliance. Although most patients make considerable efforts to attend appointments, these efforts may be overlooked within the standardized healthcare system, which prioritizes digital solutions and consumer-oriented care logic. Improving attendance requires addressing organisational and systemic barriers affecting care delivery and access, including rigid scheduling systems, hospital-initiated cancellations and rescheduling, limited coordination across departments, and inadequate information provision. Efforts to reduce missed appointments must also address digital and language inequalities, illness-related limitations and needs, and ensure that patient communication is clear, respectful, and free from stigma or blame.

## Background

Missed hospital appointments, often referred to as no-shows or non-attendance, are common across clinics worldwide. They are commonly defined as failures caused by patients who do not attend their appointments [1–3], and are widely reported to affect resource allocation, time, the quality of care, and patient outcomes [1, 3–7]. The NHS, for instance, estimated that non-attendance in general practice costs the system more than £216 million annually, directly impacting waiting times, quality of service, and patient satisfaction [8, 9]. In the USA, the cost of missed medical appointments is estimated at $150 million annually [10]. In recent years, various interventions and policies have been introduced to reduce non-attendance rates, to benefit patients and service providers alike [3, 11, 12].

In Denmark, concerns about non-attendance were formally raised in a 2004 report by the Danish Ministry of Finance, which emphasized the economic implications of non-attendance and called for intensified efforts to reduce it [13]. Subsequent national research and media reports have increasingly framed missed appointments as patient-related failures and a matter of individual compliance [2, 14–16]. In 2023, reducing non-attendance was included in a national hospital emergency plan aimed at improving hospital efficiency and productivity [17, 18].

This framing has shaped public opinion, research, and interventions. Within the Danish healthcare system, non-attendance is currently automatically registered in electronic patient records (EPRs) only for patient-related reasons, not for administrative or clinical ones [19–21]. Accordingly, national reports and registry-based studies tend to rely on pre-categorised coding schemes. They tend to present non-attendance primarily as caused by simple patient factors, or as a logistical and economic issue to be addressed through managerial instruments such as reminder systems, digital communication, incentive models, or financial penalties [2, 22–24]. They generally exclude contextual and patient-specific perspectives and, as a result, risk overlooking organisational and systemic contributions to non-attendance (i.e., “systemic blindness” [25–27]). We therefore have limited empirical insight into how institutional arrangements, communicative practices, and everyday interactions shape hospital attendance.

International research, however, indicates that reasons for non-attendance can, and often do, lie outside the patient’s control [9, 28]. Administrative errors, fragmented services, poor information provision, previous negative experience, stigma, disability, illness, social isolation, and limited health literacy are among the factors preventing patients from attending an appointment [23, 29–31]. Recent epidemiological studies have further shown that non-attendance is unequally distributed across sociodemographic groups, affecting young adults (aged 18–34) and patients with chronic health needs and mental health conditions more [20, 23]. In addition, poor communication has been documented as the sole cause of more than one in ten (>10%) patient safety incidents globally [32, 33]. At the same time, a recent Danish study demonstrated that hospital-initiated cancellations also have major consequences for patients, including worsening of physical symptoms, emotional distress, and increased hospital contacts [34, 35].

These findings call for closer attention to the systemic, organisational, and relational dynamics that shape patients’ and clinicians’ experiences of hospital encounters and access to care. This study aims to explore patients’ and healthcare professionals’ perspectives on missed hospital appointments and to identify the structural, relational, and communicative barriers and enablers of attendance, as well as potential strategies for improvement, to generate practice-oriented interventions. Rather than simply describing the sociodemographic risk factors and their correlation with non-attendance, we approach non-attendance as emerging from patients’ and healthcare professionals’ lived experiences of care, shaped by language and interaction [25, 36].

The study adopts a multilevel participatory approach. Drawing on participatory action research (PAR) and institutional ethnography [37], we engage patients, caregivers, professional interpreters, medical secretaries, clinicians, and technical specialists working with management of healthcare appointments in the Region of Southern Denmark (serving approximately 1.2 million residents) to collaboratively explore how institutional practices, communication processes, and lived experiences of care influence attendance. In doing so, we move beyond understanding non-attendance as a matter of individual compliance or choice [25]. Instead, we analyse it as embedded in structural and relational conditions, drawing attention to communicative and affective dimensions that inhibit access and participation. The article further demonstrates how participatory engagement can foster a more holistic understanding of non-attendance and inform practice-oriented improvements.

## Methods

### Study setting and design

The study was carried out in collaboration with Odense University Hospital (OUH) as part of a larger collaborative research project, involving a group of eight researchers and practitioners from the Center for Clinical Epidemiology (CKE), Department of Infectious Diseases, The Migrant Health Clinic (IMK), and the Center for Research in Patient Communication (CFPK) [38]. The data were collected between May 2023 and September 2024. During this period, we worked closely with Danish and immigrant patients, caregivers, medical interpreters, and technical and administrative support staff in the Region og Southern Denmark to identify barriers and enablers of attendance and explore solutions to reduce future non-attendance. The research team served as the project working group, planning all activities, which were carried out by individual researchers in collaboration with local administration and academic staff.

### Participatory action research (PAR)

Unlike traditional anthropological fieldwork, which emphasizes participant observation to generate deep cultural understanding, PAR actively engages researchers and participants in identifying and addressing practical problems and in developing practical solutions [37, 39–41]. This allows combining both qualitative and quantitative methods within the same framework [37, 42] and co-creating knowledge that supports inclusion, transformation, and the critical examination of existing structures and policies [43, 44].

**Fig. 1:**
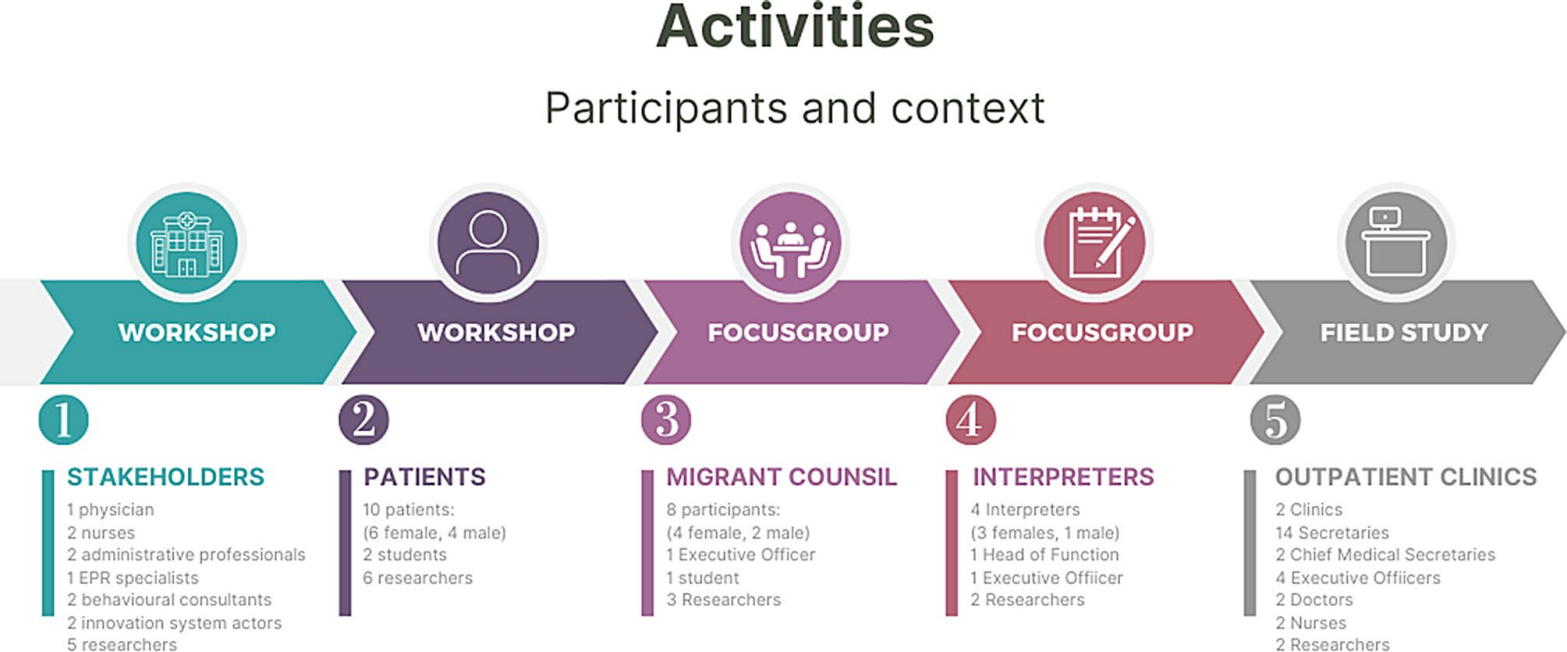
Overview of PAR activities and study participants

In this study, we combined participatory workshops [45] and focus groups [46] with ethnographic observations [47, 48] and semi-structured interviews [49, 50] to identify knowledge gaps and cover complexities and uncertainties across various settings [51]. The activities are shown in Fig. 1. They included:

- a workshop with stakeholders, representing legal and administrative bodies and healthcare services in the Region of Southern Denmark (WS)
- a workshop with patients and caregivers (WP)
- a focus group with seven immigrant patients from the Migrant Health Clinic Patient Advisory Council at OUH (FG PAC)
- a focus group with four medical interpreters in the Region of Southern Denmark (FG MI)
- a field study at orthopaedic surgery departments at two different hospitals (D1 and D2)

In addition, seven in-depth interviews with complex patients (6 female and 1 male), conducted by a master’s student (NGG) as part of her thesis, were included as supplementary empirical material [52].

### Recruitment of participants

The participants for the study were purposely recruited through professional and patient networks and on digital platforms such as LinkedIn and Facebook.

### Workshops

To identify the knowledge gap, we initiated the project with a stakeholder workshop [45]. The invited participants included clinicians, administrative healthcare professionals, digital health specialists, behavioural science consultants, health services researchers, and representatives from regional healthcare organisations. The group consisted of eight female and two male participants, aged 30 to 60. Most of the participants contacted CFPK directly after the project was announced on OUH’s webpage [53].

To gain insights into patients’ perspectives on non-attendance, we conducted a second workshop with patients and caregivers. Eight participants were recruited through advertisements on the official OUH Facebook and LinkedIn pages. All participants had prior experience with missed appointments. Two additional patients were recruited from the Patient and Relative Panel at the Center for Clinical Epidemiology. A total of six female and four male patients aged 30 to 70 participated in the workshop.

### Focus groups

In collaboration with the Migrant Health Clinic at OUH, we organized a focus group with seven members of its Patient Advisory Council (PAC) to ensure that the perspectives of patients with ethnic minority backgrounds were incorporated. The group consisted of two males and four females, aged 20 to 60, with different ethnic backgrounds, languages, diagnoses, and health histories. To validate and expand on insights into barriers faced by patients with limited Danish proficiency, we also conducted a focus group with four regional medical interpreters. The interpreters were recruited through the Region of Southern Denmark Hospital Language Service during the study period and provided services in Bosnian, Polish, Russian, and Tamil (Shri Lanka). Selection was random and determined by the Language Service based on interpreters’ availability.

All participants provided a written informed consent.

### Field study

Based on the initial mapping, the first author and a co-author (KYW) conducted a field study at two orthopaedic surgery departments (D1 and D2) located at different hospitals within the Region of Southern Denmark to understand how patient appointments and non-attendance were managed and registered in practice. Data collection included short-term ethnographic observations in each department and informal, open-ended interviews with managerial staff and medical secretaries, who played a central role in managing appointment schedules, mediating information between institutional structures and patients, and balancing relational continuity, administrative logistics, and institutional constraints. In total, 18 medical professionals participated (10 at D1 and 8 at D2; Table 1). Medical professionals were recruited in collaboration with managerial staff at each department. Consent was achieved through verbal introduction and agreement.

**Table 1:** The field study: overview of activities and participants

Activity	Department 1 (D1)	Department 2 (D2)
Initial meeting with the Chief Medical Doctor and the Chief Nurse	2	2
Interview with chief medical secretary	1	1
Observations and interviews with medical secretaries at the Department Secretariat	3	3
Observations/interviews with medical secretaries in outpatient clinic/reception	2	2
Observations of clinical professionals (physician and occupational therapist)	2	0
**Total**	**10**	**8**

### Data analysis

Workshops and focus groups were documented in real time by one or two researchers, who took notes during the discussions. Detailed written summaries were prepared immediately afterwards based on these records. Observations were documented through field notes by KYW and DS. The interviews with patients and medical professionals were audio-recorded and subsequently transcribed by KYW and NGG.

Written accounts, field notes, and interview transcripts were analysed using a reflexive thematic analysis, informed by an abductive approach [54–56]. The analysis combined a guiding interest in structural, relational, and communicative conditions shaping attendance with an open, data-driven exploration of participants’ experiences. Relevant passages referring to perceived or experienced barriers of attendance were identified manually through close readings of the material and organised into preliminary thematic categories by three authors (DS, NHG, and KYW).

All empirical material was read closely by the first author, compared, and grouped based on content similarities and the level at which they operate (individual, relational, or systemic). During this process, overlapping themes were merged and clarified to ensure coherence between analytical development and the presentation of results. Through iterative discussions within the research team, codes were compared and refined, and broader themes were developed as analytical lenses capturing barriers and enablers across participant groups and organisational contexts. Particular attention was paid to identifying similarities and differences in perspectives among patients, interpreters, administrative staff, and healthcare professionals. Emerging interpretations were subsequently discussed within the project working group and the Patient and Relative Panel at CFPK to support reflexive interpretation and ensure that the analysis resonated with user perspectives.

## Results

**Fig. 2:**
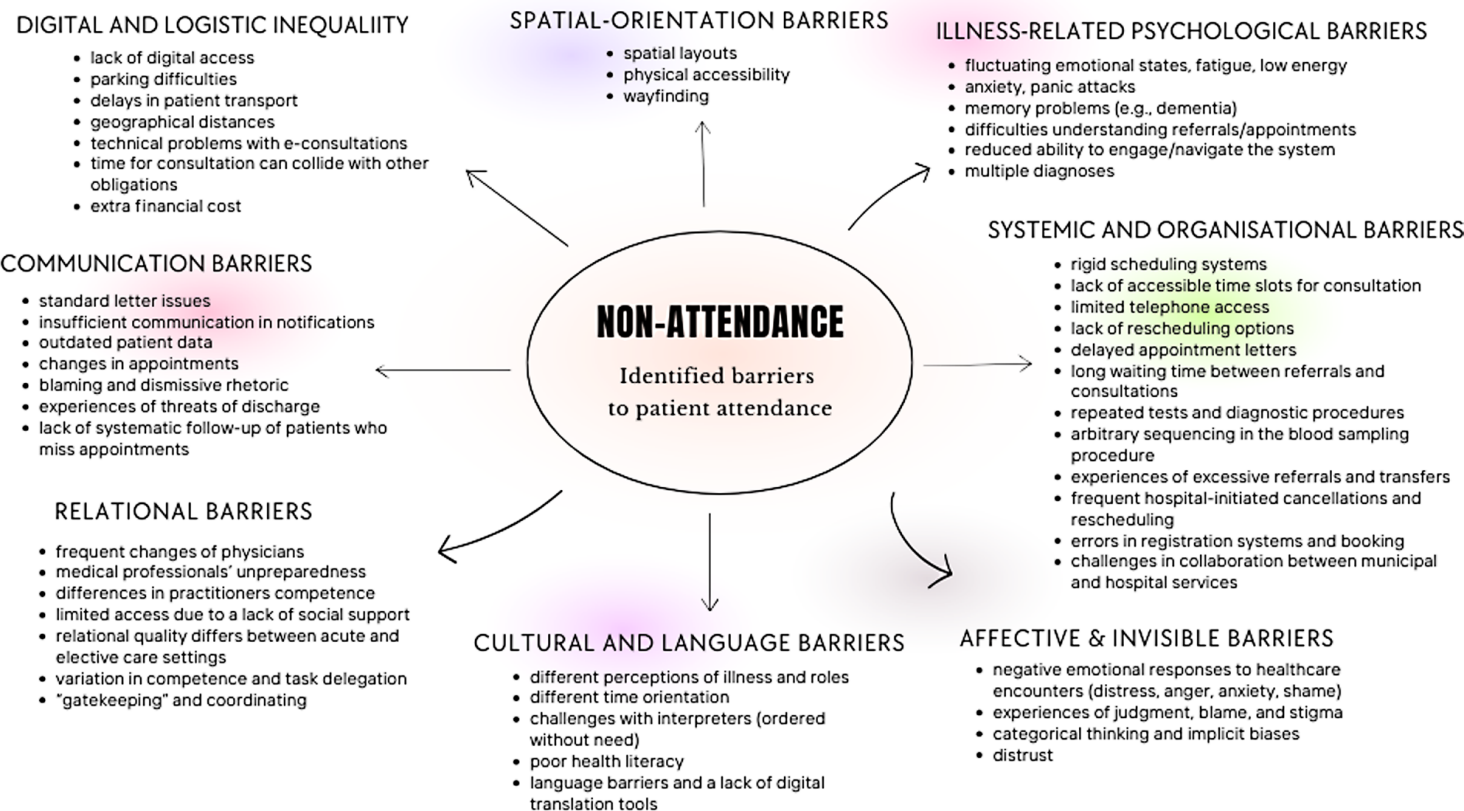
Identified themes and barriers to patient attendance

The analysis of statements from all participants (as shown in Fig. 1) identified a range of interrelated barriers to attendance, which were grouped into eight overacting themes across participant groups: (1) structural barriers related to digital and logistic inequalities; (2) systemic and organisational barriers; (3) communication barriers; (4) illness-related psychological barriers; (5) cultural and language barriers; (6) affective, or invisible barriers related to emotional burden caused by prior negative experiences of healthcare encounters, implicit biases, and categorical thinking; (7) barriers related to relational factors, such as lack of patient-provider continuity, limited social support, and loss of trust; and finally, (8) barriers related to spatial layouts of outpatient clinics and wayfinding (Fig. 2).

These eight themes emerged across perspectives from patients, professional interpreters, healthcare professionals, and administrative support staff. They highlighted limited knowledge about appointments, dismissive rhetoric, and practical challenges with digital solutions, transportation, clinic location, waiting times, appointment scheduling systems, and physical accessibility, pointing to structural and environmental conditions as key barriers to attendance. Patients additionally described longer-term emotional conditions (e.g., chronic anxiety or depressive symptoms) and psychological distress caused by negative healthcare encounters, including experiences of dismissive rhetoric and implicit biases, as important barriers to attendance.

At the same time, continuity of care and clear, direct communication emerged as key enablers of attendance in both patients’ and healthcare professionals’ accounts. A separate category concerned suggestions for reducing non-attendance. The barriers and enablers are presented below and illustrated with selected quotations from participants.

### Barriers to attendance

#### Structural barriers related to digital and logistic inequalities

Participants frequently described digital infrastructures and logistical constraints as barriers to attendance. While national digital communication systems such as digital mailboxes (e-Boks) and SMS reminders (NemSMS) were intended to streamline patient communication [57–59], they did not function equally well for all patients. Some participants reported benefitting from SMS reminders, but others lacked access to digital ID (MitID), digital mailboxes, or even a mobile phone, which prevented them from receiving appointment information effectively. One participant described the system as increasingly inaccessible:

It has become a closed system because of e-Boks. Face-to-face contact is no longer possible. (FG, PAC).

For some elderly people, socially disadvantaged individuals, and refugees, access to healthcare, mediated through complex digital infrastructures that required them to navigate a maze of multiple codes and passwords, was unavailable and required additional support. Professional interpreters reported that many refugee and immigrant patients did not receive digital letters. Yet most departments did not follow up on foreign-language patients; they lacked the correct contact details in the system or a working phone number for the patient. Secretaries expressed similar concerns about homeless people.

Geographical distance, and limited material and infrastructural resources (poor transport, limited parking, and regional regulations requiring patients from other regions to arrange and pay for their own transport) influenced attendance. It added costs and could place an additional burden on elderly patients and individuals with limited mobility or mental health issues, many of whom relied on relatives or the public on-demand transport system (Flextrafik). Patients described how long distances, unpredictable delays in Flextrafik, and the combined costs of transport and medication made attendance difficult and could lead to non-attendance. As one patient explained:

The student grant doesn’t cover a whole month – there’s no money for medication, and definitely not for transport. (FG, PAC, female, 20 years of age).

Repeated logistical barriers could further lead to a sense of futility among patients, questioning the value of scheduling appointments they were unlikely to attend:

Why try to book an appointment when you know you can’t make it anyway? (FG, PAC).

#### Systemic and organisational barriers

Non-attendance may sometimes arise from organisational constraints. Patients described both repeated diagnostic procedures and excessive referrals across services as causes of non-attendance, pointing to inefficiencies within and across care pathways. They highlighted limited scheduling flexibility, hospital-initiated cancellations and delays, restricted telephone access, long waiting times between referrals and appointments (up to several years), arbitrariness in the order of blood sampling and tests, challenges in cross-sectoral collaboration between municipal services and hospitals, and errors in registration and booking, collectively illustrating how organisational routines and system fragmentation may unintentionally contribute to missed appointments.

Several participants, for example, reported receiving short-notice appointment notifications or receiving incomplete information about the appointment location:

You receive a message in e-Boks at 4 PM saying you have to show up the next day at 8 AM – but it doesn’t say where. Then, it is a ‘no-show’. (PW).

Patients complained that last-minute notifications could also conflict with other appointments, work, or caregiving responsibilities and required additional efforts to rearrange their schedules.

Limited telephone hours and long waiting times further complicated attempts to cancel or reschedule appointments. For example, a student described difficulties contacting clinics during school hours:

I try to call and cancel during school, but the wait is so long that I have to go back to class. (FG, PAC).

Older patients and those with physical disabilities or psychiatric diagnoses (i.e., dementia or anxiety) may also struggle to meet for early appointments, but “if you try to reschedule, there is a three-month wait” (PW).

Patients also reported struggling to keep track of changing appointment times and clinical procedures, due to fragmentation and poor departmental coordination, including frequent rescheduling, duplicate tests, and overlapping appointments. This contributed to appointment fatigue and confusion about the correct appointment time:

Sometimes they change the time four times … then you end up writing down the wrong one. (Patient Workshop).

Administrative staff confirmed these challenges. Medical secretaries, for instance, described how institutional routines, limited staffing, and scheduling constraints often restricted their ability to accommodate patients’ needs. At times, administrative errors also led to patients being incorrectly marked as absent. As one participant recalled:

My husband couldn’t check in because the system listed the wrong hospital. Fortunately, he had brought the appointment letter and was seen by the doctor. (PW).

Another patient recalled being logged as a “no-show” and discharged from the clinic, even though she had called to reschedule:

It said ‘patient did not attend’—as if I hadn’t reached out at all. (PW).

#### Communication barriers

Communication issues were mentioned as another significant barrier. The key issues included insufficient information provision, unclear appointment letters, ambiguous rescheduling notices, inconsistency between information in digital letters and SMS reminders, dismissive or unempathetic rhetoric, and use of implicit or moralizing language. Many participants noted that standardised appointment letters are overly long and difficult to navigate, leading patients to feel disoriented, arrive unprepared, or end up at the wrong place. One patient recalled a letter that said:

Your appointment has been changed …”—fine, but which appointment? I have eight different ones. (PW).

Other patients admitted they struggled to identify what was important in these letters or felt insecure, particularly if appointment letters lacked clarity about time, place, and purpose, or involved a change in treating doctor. One patient also mentioned automated messages that were inadequate and difficult to interpret.

Medical secretaries and digital communication specialists also noted that communication errors sometimes originated from standardised system templates and automated letter systems that were difficult to navigate and modify, due to technical challenges (e.g., lack of an automatic search function). In some departments, secretaries had nearly a thousand templates to choose from, which increased the risk of sending the wrong letter, one with insufficient information, unclear directions, or too many attachments. “We’ve seen it [communication errors] many times”, as one of the secretaries at the orthopaedic department explained, “It’s just something the system writes, and we can’t change it from here” (D1).

Another significant factor contributing to patients’ disengagement was the use of dismissive or moralising language that resembled direct orders or scolding. Examples included comments such as:

Can’t you even figure out how to open your e-Boks?

If you didn’t call that much, we wouldn’t be so busy.

If you don’t show up, we will discharge you.

You’re an adult now, you should know how this works.

Such statements were experienced by patients as indirect accusations, disempowering, and depriving them of agency. As patients explained:

No matter what I do, I’m met with this attitude: why did you do it wrong? (PW).

Sometimes, I feel like a criminal going before a judge. Once I’ve been given the verdict, that’s it – I can’t appeal. (INT, woman, 44).

Another patient described being accused of arriving “on the wrong day” even though she followed the date stated on her appointment letter. As she explained:

“What the letter said somehow became my responsibility”, leaving her powerless and at fault. (PW).

Others experienced healthcare professionals use ironic and dismissive remarks such as “can do it, should do it” (Danish: *kan selv, skal selv*) and “you should know how it works,” or premature assurances that downplay patients’ concerns and experiences of illness, like, for example, “there’s nothing to worry about” and “these procedures are routine to us.” Such remarks, as a member of the CFPK Patient and Relative Panel, Bjarne Ledet Larsen pointed out in a presentation, “shut down the dialogue” [60]. This can impair patients’ ability to re-engage with care. As another young patient noted:

I shut down quietly because it’s a fight I can’t take. I’m not able to take it …. (INT, woman, 25).

#### Illness-related psychological barriers

Mental health conditions, cognitive challenges, and illness-related symptoms could likewise affect patients’ capacity to attend appointments. Patients living with anxiety or depression, for instance, reported that their attendance sometimes depended on how they felt on the day of the appointment. They sometimes lacked energy or had panic attacks on the day, which prevented them from attending. Others described how illness-related symptoms could affect concentration, feelings, and motivation to attend. For example, a parent of a young person with diabetes explained:

Diabetes has its own language. When blood sugar is high, the brain doesn’t function as usual. My daughter doesn’t want to go to the hospital because she doesn’t feel she is treated well, and she has developed a number of mental health issues. (FG, PAC).

Patients also described how cognitive impairment and mental health conditions (such as dementia or PTSD) could make it difficult to understand referrals, remember appointment details, or navigate the hospital system, particularly when lacking support from relatives and care professionals. As a patient with depression and anxiety noted:

Perhaps it is difficult for us to grasp the way you want us to participate. You assume your patients have a level of oversight and energy that they actually do not have (Patient Workshop).

Healthcare professionals and administrative staff also confirmed having difficulties supporting patients with dementia, cognitive impairment, or severe mental illness, particularly when patients lacked relatives or other forms of support, pointing to health-related vulnerabilities rather than pure disengagement.

#### Cultural and language barriers

Participants also described barriers related to language and cultural differences. Interpreters highlighted that differences in cultural backgrounds, education, perception of time, and familiarity with the Danish healthcare system affected patients’ expectations regarding appointments, participation in care, illness, and time management. One interpreter noted that doctors often “struggled to see things from patients’ perspectives.” They often expected patients with ethnic minority backgrounds to navigate the healthcare system with similar knowledge as the Danish patients, despite very different cultural and educational contexts. They also noted that the patient-centred consultation style common in Denmark, where patients are asked about their own expectations, could be unfamiliar to patients from non-Western backgrounds and might be perceived as useless (“just talking”). All of which could increase the risk of misunderstanding and the likelihood of no-shows.

Perceptions of time, punctuation, and attendance expectations may also differ significantly.

One interpreter described how constant delays in an outpatient clinic led patients to intentionally arrive late to their appointments, as they expected long waiting times. Such delays were sometimes recorded as no-shows.

Refugee and immigrant patients also admitted that they often struggled to understand what the letters were saying, and they often had to rely on relatives or interpreters for help. Secretaries likewise raised concerns about the Danish-only communication, emphasising that the lack of multilingual support for patients with limited proficiency in Danish (such as SMS reminders and digital letters in patients’ preferred language) required additional resources on behalf of the patient, and hence, created additional barriers for attendance:

The problem is that the messages are only in Danish, even though there are many patients now who don’t speak Danish. Appointment letters and SMS reminders are also in Danish. So, they often need someone to translate them (D1).

#### Affective, or invisible barriers

In contrast to illness-related psychological barriers, which arise from patients’ health conditions and cognitive constraints, affective barriers refer to emotional burden (feelings of anger, shame, humiliation, and being judged) emerging from negative healthcare encounters, categorical thinking, and implicit biases and stigmas. Patients described frustration and anxiety associated with healthcare appointments, repeated tests, or not being taken seriously by the healthcare professionals, describing these encounters as impersonal and routinised:

Anxiety. Afraid of not being taken seriously. The same test again and again. The smells, the lights, the sounds. The feeling of being on a conveyor belt with no compassion whatsoever, just another number going through the system in their workday. (PW).

Others described encounters with clinicians that conveyed implicit (and often inaccurate) assumptions about them, which reflected categorical thinking, contributing to feelings of being misjudged, and leading some patients to withdraw from care. One patient described how she felt mistrusted and misunderstood by a healthcare professional:

I felt she didn’t want to talk to me. I felt she had already made up her mind that I was lying. I felt genuinely violated, almost like it was an assault (INT, woman, 68).

Patients who felt judged, blamed, or misunderstood by healthcare professionals also reported avoiding calling the clinic, missing their appointments, or not returning to the clinic, because “It is an experience you do not want to repeat” (PW).

Patients also described feeling a burden on the system due to perceived time pressure and organisational routines, or “being in the way,” and “just another number,” which undermined their sense of agency and motivation to re-engage. Some patients even reported refraining from cancelling their appointments by phone to avoid this kind of attitude. One patient put it bluntly:

The healthcare system would probably run more smoothly if there were no patients at all … As a patient, you sometimes think that you just can’t take it anymore. So, you give up a little (FG PAC).

Another patient expressed this sense of resignation personally:

I’ve reached the point where I really need to have something very important before I call the hospital, because it’s like, ugh, do I have the energy to be met with that kind of attitude again, like ‘oh, here you come again, just being a burden’…. (PW).

#### Relational barriers

Disruptions and a lack of consistency in the patient-provider relationship, frequent changes in treating clinicians, and fragmented care pathways were described as barriers to attendance. Some noted that encounter quality appeared to vary between acute and scheduled care settings, while others perceived differences in clinicians’ professional competence and communication styles. When patients felt their concerns were not taken seriously or adequately understood by the doctor, they were less likely to attend their next appointment. As one patient explained:

If the doctor hasn’t taken you seriously, or hasn’t had the time to understand your illness, you do not have the strength to come again (PW).

For patients with chronic illnesses or multiple diagnoses, repeatedly having to explain their medical history to new clinicians could be emotionally burdening. As a patient with multiple sclerosis explained:

When I see a new doctor, they ask me to summarise my medical history. To which I’d think, ‘You don’t have time for that.’ That could take an hour (INT, man, 68 years, in cancer treatment).

Others described how frequent changes of treating doctors, lack of empathy, and poor quality of consultation eroded trust in the healthcare providers. For some, this contributed to feelings of not being fully seen or heard:

They don’t see us as people or listen to what you are saying. I’ve completely lost trust in them (PW).

As a result, some patients adopted coping strategies, such as postponing questions or not showing up for the appointments, until they could speak with a doctor they trusted.

Fragmented care could also foster a sense of being “tossed around”. Patients with chronic and multiple diagnoses used this expression to describe their transition from paediatric to adult care, where familiar relationships and continuity were disrupted and replaced with more fragmented care pathways. A young woman with physical disability explained:

Paediatric departments are good because there’s a nurse who knows the child … As an adult, you are moved back and forth between departments. There is no plan, and no one to help. You are tossed around. People need someone they know – a dedicated nurse or another permanent support person (FG PAC, woman, 20 years).

She pointed out that disability affects people regardless of age, which requires more permanent support to access care, “because not everyone has a parent they can turn to.”

#### Spatial orientation barriers and wayfinding

Rather than a mere information gap, non-attendance could also index breakdowns in spatial orientation (e.g., wayfinding) or institutional physical accessibility that prevent patients from fully entering the expected interactional frame of care. Although a direct effect of spatial layout on non-attendance was not conclusively established, our findings indicate that spatial organisation and patient flow within the hospital also created navigation challenges.

Accounts from patients and medical secretaries suggest that navigation difficulties and registration errors sometimes led patients to arrive at the wrong department or attend in vain. Patients were sometimes uncertain about where to report or assumed they should simply wait in the relevant area without registering their arrival. As a result, some patients remained undetected in waiting areas or moved between departments without staff being aware of their presence. Staff also noted cases where patients completed part of the pathway—for example, attending X-ray—but left the hospital before presenting at the outpatient clinic. As a result, clinical staff reported spending considerable time tracing patients across departments to check whether they had arrived, registered, or been sent to X-ray or other units. As one secretary remarked:

It becomes a bit like detective work for us to figure out where they are (D2).

During our observations, we also noticed that the spatial organisation of the clinic, reception, and waiting areas shaped interactions and opportunities for early assistance. For instance, differences in layout at D 1 and D2 influenced secretaries’ ability to identify and assist patients who appeared uncertain, disoriented, or lost. In the department where the reception area allowed clear visual contact between staff and arriving patients, staff were better able to intervene and offer guidance at arrival. In contrast, the spatial layout at the second clinic had limited visibility, reducing secretaries’ opportunities to detect and respond to such situations, and potentially increasing the risk of missed appointments.

The collective testimony, thus, underscored the multidimensional nature of missed appointments and the importance of factors beyond patient compliance.

### Enablers of attendance and opportunities for improvements

#### Continuity of care

Our findings also revealed several factors that could enable attendance. Participants’ accounts suggested that relational continuity, understood as consistent personal contact with a trusted healthcare provider, nurse, or mentor, can support attendance: It fosters positive care experiences and builds trust, thereby promoting ongoing engagement with healthcare services. Both patients and medical secretaries emphasised the importance of having a regular doctor or nurse who knew their history, highlighting relational continuity as an important condition for attendance. As one of the secretaries explains:

If patients see the same doctor, they feel more committed, and they keep their appointments (D1).

According to the medical secretaries, preference for continuity goes both ways. The doctors, too, preferred to see the same patients, and they were more reluctant to follow up with patients who did not attend their appointments if they already knew them. Medical secretaries described actively working to preserve such continuity. sometimes making discretionary scheduling decisions to meet patients’ preferences and keep patients with familiar clinicians, engaging in what sometimes has been described as optional or unrecognized labour [60, 61]. However, they often found that organisational imperatives (specialist shortages, clinician availability, emergencies, holidays, tight time-schedules, and limited scheduling flexibility) posed systemic constraints. As one secretary explained:

The doctors may simply be called away for emergency surgeries, and someone else will take over. (D1).

Patients further underscored the need for mentor support programmes and integrated care structures that accounted for patients with multimorbidity and complex care needs. The ethnic minority patients, for instance, compared the Migrant Health Clinic to the paediatric department, describing it as a “rare space of trust and attentiveness,” “a bit like the children’s clinic” (FG PAC). Thus, underscoring the need for more humane, responsive, and person-centred care structures that account for language differences, mental health, and disability-related needs.

#### Clear communication, direct interpersonal contact, and language support

In addition to care continuity, patients and medical professionals stressed the importance of clear communication, direct interpersonal contact, and multilingual language support as the important enablers of attendance, particularly for patients with ethnic minority backgrounds, limited health literacy, and low proficiency in Danish.

“People my age, they have trouble understanding what the letter says. It should be short and precise,” acknowledged a 54-year-old Somali woman. (FG, PAC).

Other patients agreed:

People are busy with e-Boks, NemID, and all the passwords, but it would be easier with a short phone call, just telling you when your appointment is (PW).

Medical secretaries and interpreters further emphasized the need for multilingual language support tools and digital solutions, including language flags in patient records, SMS reminders in patients’ preferred language, and information in multiple languages on registration and wayfinding screens. To address gaps in the system and ensure that appointments proceeded smoothly, medical secretaries frequently went beyond formal protocols and job descriptions. Instead, they relied on flexible, patient-centred practices. To support patients with language barriers, they improvised practical solutions, including ad hoc coordination, contacting relatives, and using multilingual digital tools or handwritten signs to help patients navigate clinical settings. At one clinic, for example, secretaries created a handwritten notice in Danish, English, and Arabic instructing patients to either scan their card or report to reception, to promote understanding and prevent missed appointments. They also used tools such as Google Translate when communicating with recently arrived immigrants and refugees. In this way, they compensated for organisational limitations, playing a key role in maintaining patient access and continuity of care.

#### Better digital solutions and other suggestions for improvements

The collective testimony also offered some suggestions for improvement. Apart from mentor support programmes for patients with disabilities, and language assistance tools for patients with refugee and immigrant backgrounds, proposed suggestions included:

- establishing a regular point of contact and clinician-led support for young patients
- improve clarity and customization of appointment letters and SMS reminders
- enable choice of alternative forms of communication (e.g., phone, SMS, email)
- strengthen cross-sector cooperation (e.g., between health services and educational institutions)

Several request more involvement from relatives or support persons who used to accompany patients to their visits.

Interpreters pointed to a pilot in which appointment reminders were sent in patients’ own languages, suggesting that linguistically tailored SMS reminders could improve follow-up attendance and reduce avoidable no-shows. Similarly, participants in the stakeholder workshop and hospital employees emphasized the need for better digital solutions (e.g., more reminders, improved health apps) and behavioural interventions to inform patients about the costs and consequences of a missed appointment. Their suggestions included:

- option for patients to book appointments themselves
- integration of digital appointment letters into Google Calendar
- a clear website with phone numbers and time slots for cancelling or confirming appointments
- extended telephone hours
- option to confirm or decline appointments
- improved infrastructure: signage, maps of departments, better visibility
- ensuring a clear statement of the purpose of the consultation: Why am I being called in for follow-up?
- letters, email etc need to be simplified: language, wording, and phrasing should be clearer.
- user-friendly apps, for instance, for scheduling appointments

## Discussion

Across themes, missed appointments emerged not as a single patient-level behaviour but as a multi-layered phenomenon, driven by the interplay of system complexity, logistical and technological inequity, spatial and communicative infrastructures, illness- and language-related vulnerabilities, interpersonal relationships, and a sense of disempowerment and emotional distress experienced by some patients when navigating systems that are not intuitive or accessible. According to user accounts, a significant proportion of non-attendance stems from limited accessibility, inflexibility of the scheduling system, and insufficient information provision. It may also result from patients’ responses to judgments, uncertainty, lack of continuity, and the erosion of trust following negative interpersonal and system encounters.

Thus, although we do not deny that some cases of non-attendance may stem from forgetfulness or low motivation, our findings suggest that non-attendance is rarely intentional. It may be caused by factors beyond the patients’ control, and rather reflects patients’ attempts – and failures – to navigate and fit into complex healthcare systems. The findings also indicate that direct human contact, continuity of care, and institutional flexibility can function as key enablers of attendance, which allow for better accommodating patients with special needs, who find themselves in vulnerable positions, including patients with long-term health conditions, chronic or multiple health needs, physical and mental health conditions, elderly people, and socially and ethnically marginalized individuals.

### The role of institutional structures, digitalisation, and the logic of care

Our findings suggest that framing of missed appointments as ‘non-attendance’ governs the everyday functioning of hospitals, influencing how healthcare professionals perceive problems, organize responses, speak to and relate to patients. This resonates with sociological perspectives that emphasise how the neoliberal logic of care and institutional structures shape interaction and expectations within healthcare systems [62–65]. In line with Goffman’s description of hospitals as institutional environments governed by specific standards, routines, norms, and interactional conventions [66], the collective account points to organisational priorities, such as efficiency, standardisation, and patient flow (i.e. quality standards and a production-oriented goal of moving patients efficiently through the system) as significant factors in shaping both care provision and attendance/non-attendance trajectories.

In highly standardised, efficiency-driven healthcare systems, like Denmark’s, these dynamics are reinforced by managerial and efficiency-oriented reforms that prioritise throughput and standardised workflows [25, 64]. Digital technologies, electronic records, and patient portals, introduced to strengthen patient engagement and improve care coordination by giving patients direct access to records and appointment systems, support this logic. Consequently, although active patient involvement and user-centred innovations are increasingly emphasised in research and policy agendas [67–71], in practice, the continuous proliferation of market logic, digitalisation and standardisation of care provision, and hybridization of managerial and professional roles for doctors and nurses, narrows the space for person-centred care or constructive dialogue [25]. It also shifts responsibility for attendance and health outcomes from the structural level to individual patients, potentially placing an additional burden on those least equipped to manage their care.

This logic becomes evident in everyday healthcare encounters through implicit assumptions that patients are digitally literate, can navigate complex appointment systems, and can rely on family support when needed. Yet, as our findings show, for patients who lack these resources, such expectations become barriers rather than enablers of participation.

Illness may additionally heighten patients’ sensitivity to implicit social expectations within healthcare encounters [25, 72]. When organisational norms governing care delivery no longer align with patients’ everyday realities or capacities, they may feel that the system fails to fulfil its implicit promise of care and support. It may appear inaccessible or unresponsive, creating vulnerable points in the patient journey where trust and clarity can collapse (“As a patient, you sometimes think that you just can’t take it anymore. So, you give up a little.”). For patients with chronic or life-threatening illness, physical or mental conditions, or those marginalized due to their background, language, socioeconomic status, or limited health literacy, as our findings suggest, even subtle ambiguities in appointment timing, purpose, or expectations could create additional barriers to attendance, erode trust, and trigger disengagement trajectories.

### Communication, trust, and patient engagement

Our findings further emphasise the importance of language and communication in shaping attendance. How information is conveyed, how patients are addressed, and whether communication affirms the patient’s sense of being heard and understood are critical to ensuring that the message from health professionals gets across and is acted upon. In contrast, fragmented communication, unclear messages, and dismissive or judgmental language – combined with an increased reliance on impersonal, standardised digital communication systems that do not accommodate diverse needs – can give rise to feelings of uncertainty, guilt, anxiety, or even a sense of being “on trial” (feeling like a criminal).

Consistent with previous studies [36, 73], these findings suggest that communication impacts not only whether patients understand the information, but also how they navigate care systems, respond to challenges, and feel about themselves, others, their bodies, and their illness. While language can convey emotions that invite comfort and trust [25, 74–76], our findings show that some forms of communication can generate unease or undermine patients’ sense of legitimacy and agency. For instance, routine or dismissive statements that frame serious procedures as “standard” or imply that patients should already understand how the system works could downplay the perceived seriousness of illness and weaken patients’ sense of being recognised. In such encounters, communication was experienced as closing down dialogue, making patients feel disempowered, unwanted, and uninvited. As one participant noted, such interactions could impair patients’ ability to manage their condition and reinforce negative expectations on both sides [77]. In this way, language becomes a key site where organisational logics are enacted and experienced by patients, functioning as an interface between institutional structures and patients’ lived experiences.

Digitalisation and other automated, efficiency-oriented, one-size-fits-all solutions can further displace the communicative and relational work of healthcare professionals, exacerbate inequities, and exclude patients at higher risk of non-attendance, including those with limited resources and physical and mental health conditions. In our data, both patients and staff emphasised the importance of continuity of care, direct interpersonal contact, clearer communication, and greater organisational flexibility in supporting engagement. When responsibility and organisational ownership of communication are unclear, however, accountability blurs, and the translation of institutional logics into patients’ everyday realities can falter, engendering uncertainty, fear, anxiety, and anger. For some patients, the emotional toll becomes so overwhelming that they completely withdraw from the very system designed to help them. Non-attendance, cancellation, and rescheduling, though sometimes dismissed as pragmatic responses, can therefore reflect deeper processes of relational withdrawal.

### Implications for practice

Our findings suggest that addressing non-attendance requires interventions that extend beyond individual patient behaviour and instead combine organisational adjustments with communication-focused approaches. For example, participants emphasised the potential value of multilingual communication tools, simplified appointment systems, clearer digital messaging, and direct phone contact for patients who struggle with digital platforms. Attending to material and spatial arrangements and critically examining how they co-produce the social field of action can also be relevant when addressing non-attendance, as it allows us to understand patient involvement more broadly than the information provision alone [72].

Importantly, our findings also highlight the significance of coordination work performed by frontline medical staff who actively bridge the gap between standardised organisational procedures and individual patients’ desires and needs. Supporting this relational and communicative work may therefore represent an important but often overlooked component of interventions aimed at improving attendance. As argued, beyond treatment, care entails creating an atmosphere that genuinely invites participation and strengthens communication, thereby improving both healthcare quality and attendance [72]. If the ambition is to involve patients meaningfully, we need to create conditions that allow these patients to occupy positions of agency, where they can influence the interaction and perceive themselves as competent actors. By creating such conditions, healthcare organisations may simultaneously improve patients’ experiences of the quality of healthcare services and attendance outcomes.

### Strengths and limitations

The main strength of this study lies in its ability to shed light on users’ experiences of non-attendance at outpatient appointments, foregrounding the perspectives of marginalised patients and frontline administrative staff who are often excluded from standard evaluations. The analysis reveals a connection between micro- and macro-level dynamics and illustrates how implicit language of healthcare and emotions such as anxiety, anger, guilt, and shame (including blaming and shaming practices) operate across these levels. It also documents the often-unrecognised labour undertaken by both patients and frontline administrative workers to make attendance possible and identifies some practical strategies to support future attendance. In particular, the findings emphasise how continuity and relational care can foster more meaningful and equitable engagement, pointing to institutional strategies worth reinforcing.

A potential methodological limitation of the study relates to participant self-selection. Most of the patient participants had long-standing contact with the Danish healthcare system, often due to chronic or life-threatening illness, with predominantly negative experiences of the encounters. Participants’ prior experiences with the healthcare system were likely to have shaped how they interpreted and articulated their encounters. As such, dissatisfaction and discontinuity may be disproportionately represented in the data and more visible than experiences of smooth system navigation.

In addition, the interpreter sample did not include MENAPT languages, which represent roughly one-third of the immigrant population in Denmark (56% of immigrants and their descendants originate from ten countries, the largest being Turkey, followed by Ukraine, Syria, Iraq, and Lebanon [78, 79]). Interpreters may also have brought strong professional perspectives to the topic or withheld information when it was directly related to their work, potentially influencing how certain issues were emphasised or interpreted.

To enhance the credibility and trustworthiness of the findings, we employed a multi-cited ethnographic design and engaged multiple participant groups across different institutional contexts within the PAR framework (p. 6). For example, PAC, which informed the interpretation of findings, included representatives from the MENAPT countries, while responses from medical secretaries largely aligned with those of interpreters, lending further support to several key themes. These strategies strengthen the credibility of the analysis and support the transferability of the findings to similar healthcare settings.

The researcher’s interdisciplinary background and close engagement with participants in the PAR process may also have shaped the interpretation of the findings, which we have addressed through ongoing dialogue and collective reflection within the research team.

## Conclusions

The study demonstrates that non-attendance is not solely a matter of individual choice, but a systemic and relational phenomenon shaped by structural conditions and patient-provider interaction and closely linked to how healthcare is organized and communicated. While most patients make an active effort to attend and reschedule appointments, these efforts are often constrained by barriers that require resources, flexibility, and support that not all patients have. Healthcare professionals often engage in informal labour – individual adjustments and tactics – to accommodate patients who do not fit standard pathways, which highlights both the limitations of existing systems and the potential for more responsive forms of care.

The study points to three key implications. First, improving attendance requires a shift from focusing on patient compliance to addressing structural and organisational barriers within healthcare systems. Second, communication must be recognised as central to care delivery, with greater emphasis on clarity, empathy, and cultural sensitivity to support understanding and trust. Third, continuity of care and relational support are critical, particularly for patients with complex needs or limited resources, as they help reduce uncertainty and strengthen engagement.

To conclude, PAR offers a valuable framework for collaboratively exploring non-attendance in hospital settings. It enables the identification of both barriers and enablers of attendance, reframing non-attendance from an individual behaviour issue to a systemic issue requiring shared responsibility and structural responsiveness.

These findings are relevant for healthcare practice and policy, as they highlight the need for organisational strategies that support flexible care pathways, inclusive communication, and equitable access to services. Future research should build on these insights by developing and evaluating interventions that address the systemic, communicative, and relational dimensions of missed appointments and that are more attentive to the growing number of patients with multiple health and language needs.

## Data Availability

The qualitative datasets generated and analysed in the current study are not publicly available due to their sensitive, potentially identifiable nature. Access to the data is restricted to protect participant confidentiality. De-identified data may be made available from the corresponding author on reasonable request and subject to approval by the relevant ethics committee. Note, access to data from the Danish health data authorities requires approval from the Danish Data Protection Agency. The authors of this project do not have special access privileges to the data used in this study.

## Declarations

### Patient involvement and participation

Patients and relative representatives are part of the research council at the Center for Clinical Epidemiology, and at the Centre for Research in Patient Communication, Odense University Hospital. These members have been involved in the discussion of the ideas of the study and of the study results. Patient and public representatives have not been part of the design of the study, data analyses, nor in the writing of the manuscript.

### Use of generative AI and AI-assisted technologies in the writing process

During the preparation of this manuscript, the author(s) used ChatGPT (OpenAI) to assist with grammar checking, translation of individual sentences, rephrasing, and locating some supplementary sources for background context. Canva templates were used for data visualization. No AI tools were used for data analysis or to generate substantive content of the article. These were carried out manually by the author(s). All AI-assisted outputs were critically reviewed and edited by the author(s), who take full responsibility for the final content of the manuscript.

## Abbreviations

CFPK: Centre for Research in Patient Communication
CKE: Center for Clinic Epidemiology
D1: Department 1
D2: Department 2
DNA: Did Not Attend
E-Box: Digital Post App
EPR: Electronic patient record
FG: Focus group
Flextrafik: A public on-demand transport system for individuals with limited mobility or without access to regular transit
ForSa-P: Center for Forskning Sammen med Patienter og Pårørende (Center for Research Together with Patients and Relatives)
MENAPT: Middle East, North Africa, Pakistan, and Turkey
MIK: Migrant Health Clinic
MitID: Danish digital identification system
NemSMS: the SMS reminder service
NHS: UK National Health Service
OUH: Odense University Hospital
PAC: Patient Advisory Council
PAR: Participatory Action Research
WP: Workshop with Patients
WS: Workshop with Stakeholders
INT: Interviews
